# Release of HMGB1 in Podocytes Exacerbates Lipopolysaccharide-Induced Acute Kidney Injury

**DOI:** 10.1155/2021/5220226

**Published:** 2021-09-27

**Authors:** Zhao Gao, Li Lu, Xinghua Chen

**Affiliations:** ^1^Department of Nephrology, Xiangyang Central Hospital, Hubei University of Arts and Science, Xiangyang, 441000 Hubei, China; ^2^Department of Nephrology, Renmin Hospital of Wuhan University, 238 Jiefang Rd., Wuhan, 430060 Hubei, China

## Abstract

**Objective:**

Acute kidney injury (AKI) usually occurs during sepsis. Inflammation factors, such as high-mobility group box 1 (HMGB1), are dramatically upregulated under septic conditions. In our current work, the functions of HMGB1 in AKI were explored.

**Methods:**

An AKI model was induced by the lipopolysaccharide (LPS) challenge in C57 mice. Podocytes were challenged by LPS for different durations. Subsequently, podocytes transfected with HMGB1 siRNA were exposed to LPS for 24 h. The expressions of supernatant HMGB1 and cellular active caspase-3 were examined by Western blotting analysis. To explore the effect of HMGB1 on tubular epithelial cells (TECs), HK-2 cells were exposed to HMGB1 at various concentrations for 24 h. Epithelial-mesenchymal transition (EMT) of HK-2 cells was evaluated by Western blotting analysis. Mitochondrial division and apoptosis of HK-2 cells were assessed by MitoTracker Red and Western blotting analysis, respectively.

**Results:**

Compared with the sham control group, the expression of HMGB1 was increased in the kidney of AKI mice. Moreover, the expression of supernatant HMGB1 was increased in LPS-challenged podocytes compared with the control group. Knockdown of HMGB1 attenuated LPS-induced podocyte injury. Besides, EMT in TECs was triggered by HMGB1. Mitochondrial damage and apoptosis of HK-2 cells exposed to HMGB1 were markedly elevated compared with the control group.

**Conclusions:**

Collectively, HMGB1 release in podocytes was induced by LPS, subsequently leading to exacerbated AKI.

## 1. Introduction

Sepsis-related endotoxemia remains the main cause of acute kidney injury (AKI) [1]. Patients with septic AKI have a higher mortality rate compared with nonseptic AKI patients [2]. Endotoxemia is mostly caused by lipopolysaccharide (LPS), which is released from Gram-negative bacteria. Endotoxemia can induce systemic inflammatory activation and lead to AKI [3].

LPS binds to toll-like receptors (TLRs) to activate immune cells, leading to the release of high-mobility group box 1 (HMGB1). When HMGB1 interacts with receptors for advanced glycation end products (RAGE) and TLRs, proinflammatory factors are released, which exacerbates tissue damage [4–6].

Podocytes, as a terminally differentiated cell type, constitute the glomerular filtration barrier in the kidney. Podocytes have a fundamental function in maintaining the normal structure and performance of the kidney [7–9]. Recent studies have reported that LPS induces podocyte damage and promotes the progression of AKI [6, 10]. However, the roles of HMGB1 released by damaged podocytes in sepsis-associated AKI remain largely undetermined.

In previous studies [11–13], renal tubular epithelial cells (TECs) are the focus of mainstream research in AKI. Therefore, the role of glomerular cells including podocytes in AKI is poorly studied. In our current work, we aimed to assess the impacts of LPS-triggered HMGB1 release in podocytes on TECs.

## 2. Materials and Methods

### 2.1. Animal Model

Male C57 mice (7 weeks old, weighing 20–22 g) were obtained from the Center of Experimental Animals of Wuhan University (Hubei, China) and housed in a pathogen-free environment under controlled conditions, including temperature and humidity. After 1 week, mice were stochastically divided into two groups as follows: control group and LPS group. Mice in the control group (*n* = 16) were administered with saline via intraperitoneal injection. Mice in the LPS group (*n* = 16) were administered with LPS (Escherichia coli 055:B5 Sigma, 10 mg/kg) through the same way. Mice were sacrificed at 24 h (*n* = 8) and 48 h (*n* = 8) after LPS injection. The blood and kidneys were collected for further study. The experimental procedure was authorized by the Ethical Committee of the Renmin Hospital of Wuhan University.

### 2.2. Cell Culture and Treatment

Mouse podocyte cell line was gifted by Dr. Peter Mundel (Massachusetts General Hospital, Boston, MA) and cultivated in RPMI-1640 medium (HyClone, USA) containing 10% fetal calf serum (FCS, Gibco, USA) and 10 U/mL recombinant murine interferon-*γ* (PeproTech, Rocky Hill, NJ) in a Thermo Fisher incubator (Marietta, OH, USA) at 33°C. When the cell confluence reached 80%, podocytes were cultivated in the absence of interferon-*γ* at 37°C to induce differentiation. The differentiated podocytes were exposed to LPS (1 *μ*g/mL) for various durations [10].

Human TEC cell line (HK-2 cells) was cultivated in DMEM/F12 (HyClone, Rockville, MD, USA) supplemented with 10% FCS at 37°C. HK-2 cells were stimulated with gradient concentrations of HMGB1 (Abcam, 0, 0.1, 1, and 10 *μ*g/mL) for 24 h. Each experiment was performed in triplicate.

### 2.3. HMGB1 RNA Interference

HMGB1 siRNA was transfected to podocytes according to the HiPerfect Transfection Reagent guide (QIAGEN, Germany). Briefly, podocytes were seeded in 6-well plates at a density of 2 × 10^5^ cells per well. Subsequently, scramble RNA or 10 nmol/L HMGB1 siRNA (QIAGEN, Germany) and HiPerfect Transfection Reagent were added to each well. The cells in 6-well plates were incubated at 37°C for 48 h.

### 2.4. Blood Biochemical Analysis

Blood samples were collected by cardiac puncture under isoflurane anesthesia. Kidneys were infused by cold PBS and then promptly isolated for histopathological examination and Western blotting analysis. Levels of renal functional biomarkers (blood urea nitrogen (BUN) and serum creatinine (SCr)) were examined at the Renmin Hospital of Wuhan University.

### 2.5. Ultrafiltration Concentration of Cell Culture Supernatant

Amicon Ultra-4 centrifugal filter devices (Millipore) were used to perform ultrafiltration concentration of the cell culture supernatant based previous methods [14] and the product manual. Briefly, 4 mL cell supernatant was transferred into Amicon Ultra-4 centrifugal filter devices. Subsequently, the cell supernatant was centrifuged at 3,000 g for 30 min at 4°C. After centrifugation, 100-150 *μ*L concentrated supernatant was used for Western blotting analysis.

### 2.6. Immunohistochemistry (IHC)

Paraffin-embedded sections were used for IHC staining. The sections were incubated with primary antibody against HMGB1 (1 : 1,000, Abcam) at 4°C overnight. Subsequently, the sections were rinsed with PBS three times, followed by incubation with secondary antibody (Dako, USA) at 37°C for 30 min. The pictures were analyzed using Image-Pro Plus 5.10 (Media Cybernetics, USA).

### 2.7. Western Blotting Analysis

Total proteins were isolated from the kidneys or cells. Equal amounts of proteins were subjected to sodium dodecyl sulfate-polyacrylamide gel electrophoresis (SDS-PAGE) and then electrotransferred onto PVDF membranes. The blots were incubated at 4°C overnight with primary antibodies against HMGB1 (1 : 1,000, Abcam), active caspase-3 (1 : 1,000, Abcam), E-cadherin (1 : 1,000, Proteintech), *α*-SMA (1 : 1,000, Abcam), actin (1 : 1,000, Abcam), and tubulin (1 : 1,000, Abcam). Subsequently, the blots were rinsed with PBS three times, followed by incubation with Alexa Fluor 790 IgG (1 : 30,000, Jackson ImmunoResearch, West Grove, PA, USA) at 37°Cfor 1 h. Immunoreactive bands were visualized using the Odyssey Infrared Imaging System (Lincoln, NE, USA).

### 2.8. Examination of Mitochondrial Fragmentation in HK-2 Cells

MitoTracker Red (Invitrogen, USA) was used to evaluate mitochondrial morphology in HK-2 cells. The cell climbing films were stained with 500 nM MitoTracker Red at 37°C for 30 min in a dark room. Subsequently, the films were rinsed with PBS three times in the dark and observed using an Olympus confocal microscope (Tokyo, Japan).

### 2.9. Statistical Analyses

Data were presented as means ± SEM and analyzed using GraphPad Prism 5.0. *t*-test or one-way analysis of variance was adopted to compare differences in various groups. *P* < 0.05 was regarded as statistically significant.

## 3. Results

### 3.1. AKI Is Induced by LPS *In Vivo*

**Figure 1:**
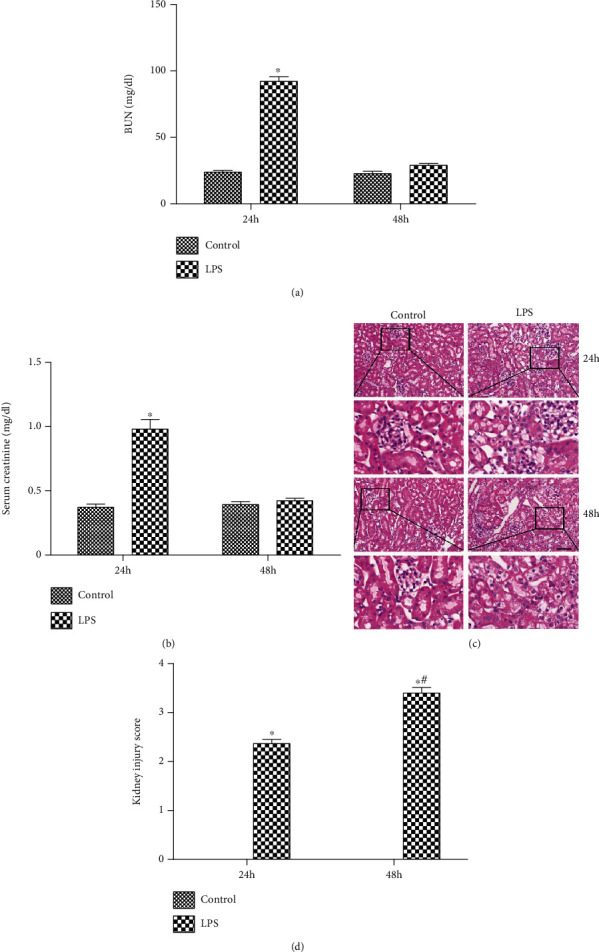
AKI is induced by LPS. (a) The level of BUN in both groups after the LPS challenge. (b) The level of SCr in both groups after the LPS challenge. (c) Representative HE staining in both groups after the LPS challenge. (d) Representative Kidney injury score in both groups. The black arrow indicated injured tubules. Scale bars 50 *μ*m. ^∗^*P* < 0.05 compared with the control group; ^#^*P* < 0.05 compared with the LPS group in 24 h.

After the LPS challenge for 24 h, mice in the LPS group became inactive and developed diarrhea, increased eye secretions, and lethargy. The levels of BUN and SCr in the LPS group (92.52 ± 9.40 mg/dL and 0.95 ± 0.21 mg/dL, respectively) were also significantly increased compared with those in the control group (24.67 ± 4.35 mg/dL and 0.39 ± 0.08 mg/dL, respectively) at 24 h postchallenge (Figure 1(a)). Thereafter, the levels of BUN and SCr in the LPS group were decreased, and there was no statistical difference compared with the control group at 48 h postchallenge (Figure 1(b)). Histopathological examination exhibited swelling, dilatation, vacuolization, and detachment of TECs (Figure 1(c)). Kidney injury underwent semiquantified analysis by counting the percent of injured tubules and scored as follows: 0 = none, 1 ≤ 10%, 2 = 11 − 25%, 3 = 26 − 45%, 4 = 46 − 75%, and 5 ≥ 76%, as per the previous protocol [15]. Although the levels of BUN and SCr in the LPS group were decreased after 48 h, kidney injury score in LPS groups at 48 h was significantly increased compared with the LPS groups at 24 h(Figure 1(d)).

### 3.2. HMGB1 Is Upregulated in the Kidney of AKI Mice

**Figure 2:**
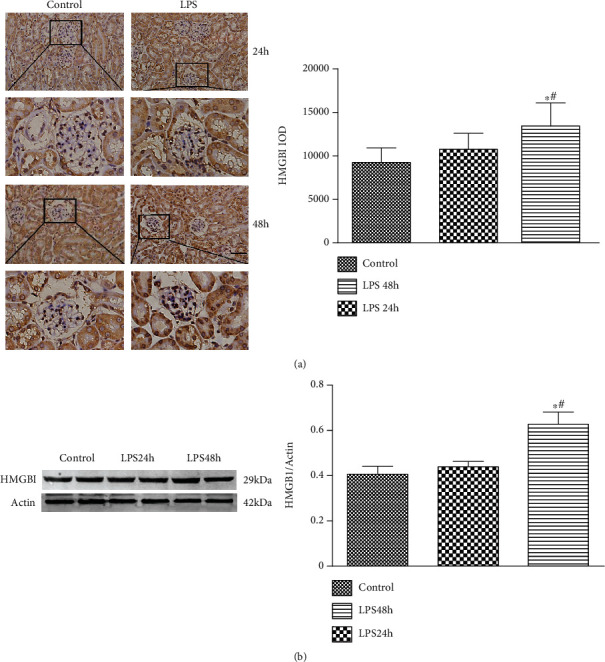
The level of HMGB1 is increased in the kidney of AKI mice. (a) Representative IHC image of HMGB1 expression in both groups after the LPS challenge. (b) Representative Western blotting analysis image of HMGB1 expression in both groups after the LPS challenge. Scale bars 50 *μ*m. ^∗^*P* < 0.05 compared with the control group; ^#^*P* < 0.05 compared with LPS 24 h group.

IHC examination was adopted to detect the level of HMGB1 in both groups.  Figure 2(a) shows that the expression of HMGB1 was mildly increased in glomeruli of LPS group at 24 h postchallenge. Subsequently, the expression of HMGB1 was significantly increased in the LPS group at 48 h postchallenge compared with the control group. Besides, Western blotting analysis revealed that after the LPS challenge, the level of renal HMGB1 was elevated at 24 h postchallenge and remarkably enhanced at 48 h postchallenge, which was consistent with IHC examination (Figure 2(b)).

### 3.3. Release of HMGB1 in Podocytes Is Increased upon Exposure to LPS

**Figure 3:**
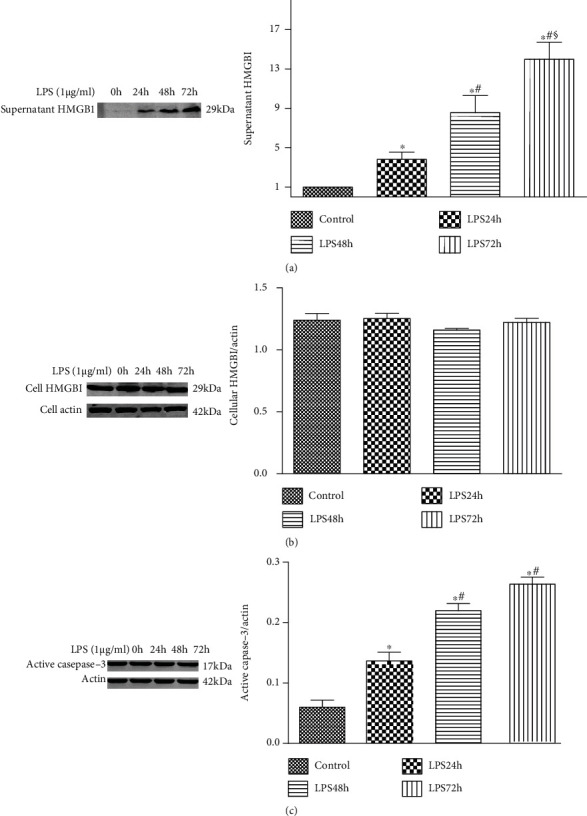
HMGB1 expression is increased in podocytes exposed to LPS. (a) Representative Western blotting analysis of supernatant HMGB1 expression after exposure to LPS for different durations (0, 24, 48, and 72 h). (b) Representative Western blotting analysis of cell HMGB1 expression after exposure to LPS for different durations (0, 24, 48, and 72 h). (c) Representative Western blotting analysis image of active caspase-3 expression after exposure to LPS for different durations (0, 24, 48, and 72 h). ^∗^*P* < 0.05 compared with the control group; ^#^*P* < 0.05 compared with the 24 h group; ^§^*P* < 0.05 compared with the 48 h group.

The levels of supernatant HMGB1 were gradually increased along with the LPS exposure. However, no statistical difference in the cellular HMGB1 was detected between the two groups (Figure 3(a)). Usually, the active caspase-3 is used to represent the apoptosis level.  Figure 3(b) shows that the level of active caspase-3 was significantly enhanced at 24 h postchallenge. However, no statistical difference was found between the 48 h group and the 72 h group.

### 3.4. Knockdown of HMGB1 Attenuates LPS-Induced Podocyte Injury

**Figure 4:**
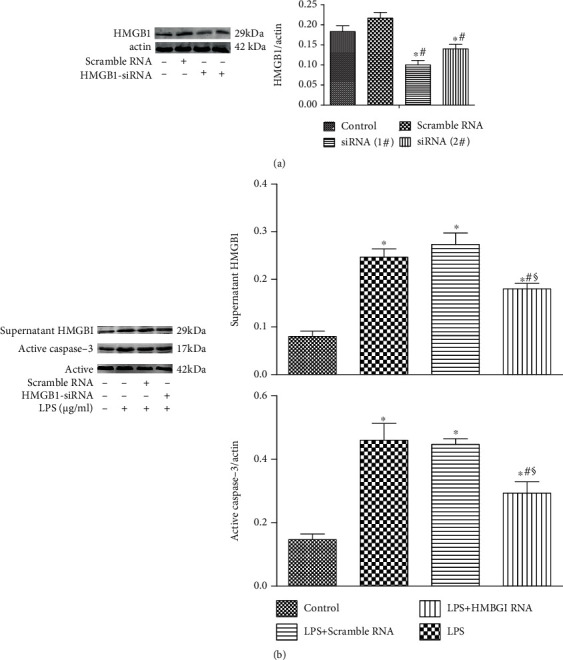
Knockdown of HMGB1 attenuates LPS-induced podocyte injury. (a) Representative Western blotting analysis of HMGB1 expression in podocytes after HMGB1 siRNA transfection. (b) Representative Western blotting analysis image of supernatant HMGB1 and active caspase-3 expression in podocytes transfected by HMGB1 siRNA after exposure to LPS for 24 h. ^∗^*P* < 0.05 compared with the control group; ^#^*P* < 0.05 compared with HMGB1 group; ^§^*P* < 0.05 compared with the scramble group.

To investigate the role of HMGB1 in podocytes, HMGB1 expression in podocytes was suppressed by HMGB1 siRNA (Figure 4(a)).  Figure 4(b) reveals that LPS promoted the levels of supernatant HMGB1, but knockdown of HMGB1 significantly decreased the LPS-induced levels of supernatant HMGB1. Moreover, knockdown of HMGB1 decreased the LPS-induced upregulation of active caspase-3 (Figure 4(b)). These results indicated that LPS-induced podocyte injury was suppressed by HMGB1 siRNA.

### 3.5. Epithelial-Mesenchymal Transition (EMT) in HK-2 Cells Is Induced by HMGB1

**Figure 5:**
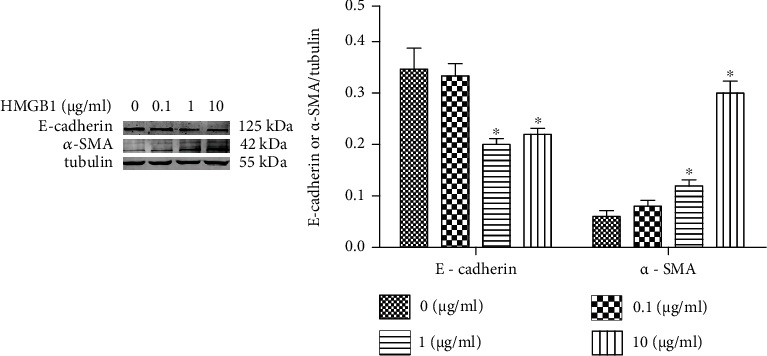
EMT of HK-2 cells is induced by HMGB1 .The HK-2 cells were challenged with HMGB1 at various concentrations (0, 0.1, 1, and 10 *μ*g/mL) for 24 h. Representative Western blotting analysis image of *α*-SMA and E-cadherin expressions in different groups. ^∗^*P* < 0.05 compared with the control group.

To determine the effect of HMGB1 on EMT in HK-2 cells, E-cadherin and *α*-SMA were analyzed by Western blotting.  Figure 5 shows that the expression of E-cadherin was significantly reduced following the HMGB1 (1 *μ*g/mL) treatment. Consistently, the expression of *α*-SMA was significantly increased following the HMGB1 (1 *μ*g/mL) treatment. These results indicated that EMT was induced by HMGB1 in HK-2 cells.

### 3.6. Mitochondrial Damage in HK-2 Cells Exposed to HMGB1

**Figure 6:**
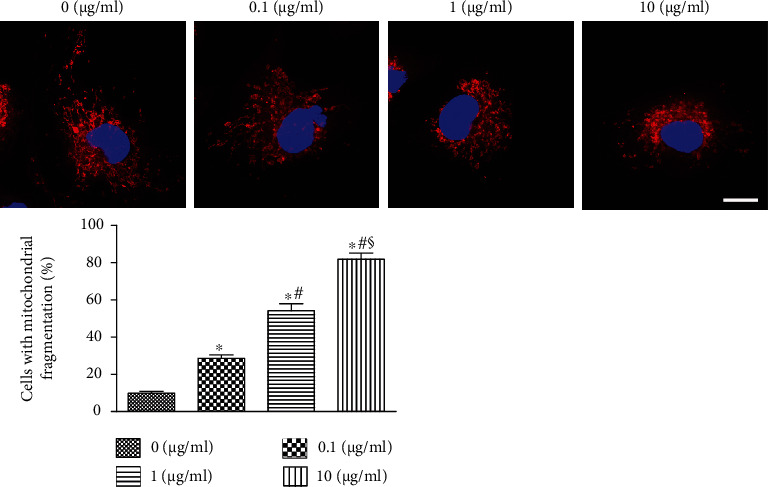
Mitochondrial damage in HK-2 cells challenged by HMGB1. The confocal microscope shows mitochondria stained with MitoTracker Red. The proportion of cells with mitochondrial fragmentation was evaluated. Scale bars 10 *μ*m. ^∗^*P* < 0.05 compared with the 0 *μ*g/mL group; ^#^*P* < 0.05 compared with the 0.1 *μ*g/mL group; ^§^*P* < 0.05 compared with the 1 *μ*g/mL group.

MitoTracker Red was used to evaluate mitochondrial morphology of HK-2 cells. Usually, mitochondrial morphology of cells was filamentous or fragmented. For cells with mixed mitochondrial morphology, the mitochondrial morphology of the cells was based on the majority (>70%) of mitochondria. More than 100 cells per dish were evaluated at several stochastic fields [16]. Mitochondria were stained by MitoTracker Red, showing red fluorescence.  Figure 6 shows that the mitochondria in the control group exhibited filamentous and network-shaped morphology. However, the mitochondria became fragments during HMGB1 treatment. These results showed that the mitochondrial structure was damaged after HMGB1 treatment.

### 3.7. The Apoptosis of HK-2 Cells Is Increased when Exposed to HMGB1

**Figure 7:**
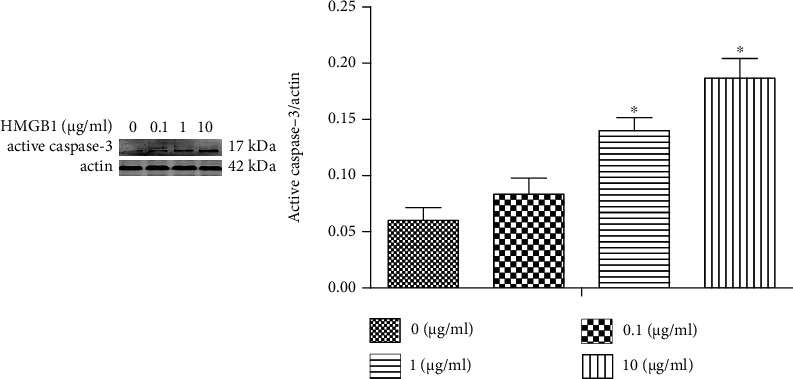
HK-2 apoptosis is increased once exposed to HMGB1. The HK-2 cells were challenged by HMGB1 at various concentrations (0, 0.1, 1, and 10 *μ*g/mL) for 24 h. Representative Western blotting analysis of active caspase-3 expression in different groups. ^∗^*P* < 0.05 compared with the control group.

Active caspase-3 was used to determine the apoptosis of HK-2 cells when challenged by HMGB1. The apoptosis in HK-2 cells was gradually increased upon the HMGB1 exposure compared with the control group (Figure 7).

## 4. Discussion

In our previous study, we have investigated the impacts of hypoxia on AKI [17]. In our current work, the role of inflammation in AKI was investigated. Accumulating evidence suggests that ischemic/hypoxic conditions, nephrotoxic agents, and inflammation are related to the development of AKI. Sepsis accounts for 45–70% of all causes of AKI, and the related mortality in septic AKI patients is higher compared with all other AKI categories [18]. HMGB1 belongs to a high-mobility protein group and plays an important role in mediating the cellular inflammation. HMGB1 can specifically bind to TLR-4 and promote the secretion of inflammatory cytokines [19]. In our current study, the role of HMGB1 in AKI was investigated.

As a bacterial endotoxin, LPS can enter into the circulation system and activate an inflammatory response, eventually leading to AKI [20]. Currently, the sepsis-associated AKI model has been established by LPS to explore the roles of HMGB1 in the pathogenesis of AKI. In our current work, the levels of serum Cr and BUN in the LPS group were significantly increased. Histopathological examination revealed swelling, dilatation, vacuolization, and detachment of TECs, indicating AKI. Besides, the expression of HMGB1 in the kidney of the LPS group was marked elevated compared with that of the control group, indicating that HMGB1 played an important role in AKI.

Recently, Huang et al. [6] have found that inhibition of HMGB1/TLRs/NF-*κ*B signaling alleviates LPS-induced apoptosis and inflammatory response of podocytes. Besides, Jin et al. [21] have shown that inhibition of HMGB1 attenuates diabetic nephropathy serum-induced apoptosis and EMT of podocytes. To investigate the role of podocytes in AKI, a mouse podocyte cell line was challenged by LPS. The levels of supernatant HMGB1 were gradually increased along with LPS treatment. Although the level of active caspase-3 was significantly enhanced at 24 h postchallenge, no statistical difference was found between the 48 h group and the 72 h group. The above-mentioned findings suggested that HMGB1 was mainly secreted by podocytes and maybe partly released by apoptotic podocytes. Previous studies have shown that HMGB1 is a late inflammatory factor and secreted by immune cells and injured cells [22, 23]. To investigate the role of HMGB1 in podocytes, HMGB1 expression in podocytes was suppressed by HMGB1 siRNA. Knockdown of HMGB1 significantly decreased the LPS-induced levels of supernatant HMGB1 and the levels of active caspase-3. These results indicated that knockdown of HMGB1 could attenuate LPS-induced podocyte injury.

To investigate the effect of HMGB1 on TECs, we evaluated EMT, mitochondrial damage, and apoptosis in the present study. We found that EMT, mitochondrial damage, and apoptosis of TECs exposed to HMGB1 were dramatically elevated compared with the control group, indicating that HMGB1 could induce injury and apoptosis of TECs. A previous study has found that melatonin promotes the proliferation of TECs and improves the cell cycle arrest via inhibiting HMGB1 [24]. A previous study has verified that HMGB1 and LPS could induce TEC mitochondrial dysfunction, inflammation, and stress [25].

Obviously, there are some limitations in our research. Firstly, it is not performed to inhibit the expression of HMGB1 in vivo. Secondly, it is unclear on how HMGB1 is secreted into the extracellular space. Therefore, it is necessary and urgent to investigate the precise mechanism of HMGB1 in AKI in the future.

## 5. Conclusions

LPS induced the expression of HMGB1 in the kidney. HMGB1 release in podocytes was triggered by LPS. Knockdown of HMGB1 attenuated LPS-induced podocyte injury. Subsequently, HMGB1 promoted TEC EMT, mitochondrial damage, and apoptosis of TECs, leading to exacerbated AKI. This study maybe helps us to clarify the molecular mechanism of septic AKI and to supply the prevention means.

## Data Availability

All data of this study are included in the article.
